# Dose-Response Association of Tai Chi and Cognition among Community-Dwelling Older Adults: A Systematic Review and Meta-Analysis

**DOI:** 10.3390/ijerph18063179

**Published:** 2021-03-19

**Authors:** Mei-Lan Chen, Stephanie B. Wotiz, Starr M. Banks, Sabine A. Connors, Yuyin Shi

**Affiliations:** 1 Byrdine F. Lewis College of Nursing and Health Professions, Georgia State University, Atlanta, GA 30303, USA; stephaniewotiz@yahoo.com (S.B.W.); starrbanks@gmail.com (S.M.B.); sabineconnors@gmail.com (S.A.C.); 2 Gerontology Institute, Georgia State University, Atlanta, GA 30303, USA; 3 Department of Mathematics, University of Maryland, College Park, MD 20742, USA; yshi919@umd.edu

**Keywords:** Tai Chi, cognitive function, exercise, dose–response relationship, cognitive impairment, older adults, meta-analysis, PRISMA statement guideline

## Abstract

Previous studies indicated that Tai Chi might be an effective way to improve or prevent cognitive impairments in older populations. However, existing research does not provide clear recommendations about the optimal dose of Tai Chi practice, which is the most effective in improving cognitive function in older adults. The purpose of this systematic review and meta-analysis was to investigate the dose–response relationship between Tai Chi and cognition in community-dwelling older adults. A total of 16 studies with 1121 subjects were included in this study. Meta-regression analyses of Tai Chi duration (Tai Chi session duration, Tai Chi practice duration per week, study duration, and Tai Chi practice duration for the entire study) on the study effect size (ES) were performed to examine the dose–response association of Tai Chi and cognition. The results showed that there was a positive effect of Tai Chi on cognitive function, but there were no statistically significant dose duration effects on cognition. The findings suggest that Tai Chi has beneficial effects on cognitive function, but a longer duration was not associated with larger effects. In order to establish evidence-based clinical interventions using Tai Chi, future research should clearly demonstrate intervention protocol, particularly the style and intensity of Tai Chi.

## 1. Introduction

One significant burden for older adults and their family is age-related cognitive impairments. Worldwide, about 50 million people have dementia [1]. In 2020, nearly 5.8 million older people in the United States were living with Alzheimer’s disease, the most common type of dementia, and the total number is projected to escalate to 14 million older adults by 2050 [2]. Alzheimer’s disease is the 6th leading cause of death among American adults and the 5th leading cause of death in older adults [2]. By 2040, an estimated cost of treating Alzheimer’s disease is projected to be $379–511 billion [3]. Cognitive impairments and dementia can cause substantial disability, reduce health-related quality of life, and increase health care cost [1,2,4]. Therefore, maintaining cognitive health is a critical issue for older adults and their communities.

Currently, there is no known cure for dementia or Alzheimer’s disease. Thus, developing non-pharmacological interventions to improve cognitive function or delay the onset of dementia for older adults is essential [1]. Physical activity interventions may have the potential protective effect on cognitive function, such as Tai Chi [1,2,5,6,7,8]. Tai Chi is an Eastern form of multilevel mind–body exercise with the characteristics of aerobic exercise, strength, and flexibility trainings. The practice of Tai Chi involves physical, psychosocial, cognitive, and meditative skills. The components of Tai Chi intervention include meditation, attention and rhythmic breathing training, learning of movement patterns, and mobility and postural control training [5,6,7,8]. Current literature indicates that Tai Chi has positive effects on cognitive function among older adults [5,6,7,8]. For example, Taylor-Piliae et al. (2010) found that older adults engaging in Tai Chi had significant improvements on cognition as compared with the Western exercise or no exercise [8]. Similarly, the older adults who participated in Tai Chi in the study by Tao et al. (2016) had greater improvements on memory quotient and hippocampal function and connectivity as compared with the control group [7]. These studies suggest that Tai Chi may mitigate cognitive decline in older adults.

Previous systematic reviews and/or meta-analyses also indicated that Tai Chi might be an effective way to improve memory performance or prevent cognitive impairments in older populations [9,10,11,12,13]. However, there has not been a systematic review and meta-analysis examining the dose–response effect of Tai Chi on cognitive performance in the population of older adults. In order to establish evidence-based clinical interventions using Tai Chi, practitioners require recommendations of effective dosage. Yet, existing research does not provide clear recommendations about the optimal dose of Tai Chi practice, which is the most effective in improving cognitive function in older adults. The dose–response effects of Tai Chi on cognition remain unknown. To fill this gap, the purpose of this systematic review and meta-analysis was to investigate the dose–response relationship between Tai Chi practice and cognitive function in community-dwelling older adults.

## 2. Materials and Methods

### 2.1. Search Strategy

A systematic review and meta-analysis was conducted in accordance with the preferred reporting items for systematic reviews and meta-analyses (PRISMA) statement guideline [14]. Both computerized and hand-searching strategies were used in this study. For the computerized search, electronic research literature searches of the PubMed, CINAHL, Cochrane Library, EMBASE, SPORTDiscus, PsychInfo, and Ageline databases were performed. The key words and search terms used for a thorough literature search via search engines were “Tai Chi” and “Tai Ji”. No language, year of publication, and/or geographic location preferences were applied. For the hand searches, the reference lists from all relevant reviews and meta-analyses were additionally searched for peer-reviewed papers not found with the computerized search.

### 2.2. Inclusion and Exclusion Criteria

Studies were included in this systematic review and meta-analysis if they met all of the following criteria: (a) research participants were community-dwelling older adults, (b) all subjects in a study were 55 years old or older, or the average subject age was ≥60 years, (c) participants in the Tai Chi group received a quantifiable dose of Tai Chi, (d) participants in the control group received either health education or no intervention, (e) subjects had normal cognition (or had no reported cognitive impairment), or mild cognitive impairment (MCI), (f) outcomes related to cognitive function were measured, and (g) study reports were written in English or Chinese, or translated into English. Studies were excluded for the following criteria: (a) subjects were institutionalized or receiving long-term care in a hospital or nursing home, (b) study focused on persons with chronic disease or health condition, (c) participants in the control group received exercise or other physical activity interventions, or (d) insufficient data were reported in the study to calculate the total or per session dose of Tai Chi, or an effect size. Prior to excluding any studies for insufficient data, the present study’s authors attempted to retrieve the necessary data by contacting the corresponding author via email and/or ResearchGate.

### 2.3. Screening and Selection Strategy

The inclusion and exclusion criteria described above were strictly applied to the study screening and selection process. The study selection process was performed independently by at least two reviewers. Study reports written in Chinese were reviewed by two bilingual Chinese–English reviewers. All reviewers are the present study’s authors. Any disagreements between the two reviewers were solved through arbitration or discussion with a third investigator.

### 2.4. Data Extraction

Two authors independently reviewed and extracted necessary data from the selected studies. Any disagreements were resolved through mediation with a third author or by contacting corresponding author of the study via email. The Tai Chi dosages were extracted from each study in the following 4 types: Tai Chi session duration (minutes), Tai Chi practice duration per week (including warm-up and cool-down; minutes), study duration (weeks), and Tai Chi practice duration for entire study (minutes). For cognitive outcome measurements, cognitive functions were categorized into five domains, including global cognition, executive function, language, learning and memory, and spatial and quantitative. Cognition data were extracted in the forms of means, standard deviations (SD), and sample sizes for both the Tai Chi and control groups in all studies except in the studies by Man et al. (2010), Mortimer et al. (2012), Tao et al. (2016), and Taylor-Piliae et al. (2010) [7,8,15,16]. Of these latter studies, the Man et al. (2010) study reported the mean difference between Tai Chi and control groups, sample sizes, and related *p*-value, whereas the remaining studies used mean differences, SD of differences, and sample sizes from both the Tai Chi and control groups [15]. These descriptors were reported as either post-test only or as changes from pretest to post-test. For any insufficient data or missing data, the present study’s authors contacted the corresponding authors via email and/or ResearchGate.

### 2.5. Assessment of Methodological Quality

The Effective Public Health Practice Project (EPHPP) Quality Assessment Tool for Quantitative Studies [17] was used to examine the methodological quality of the included studies. The EPHPP Quality Assessment Tool evaluates six components including selection bias, study design, confounders, blinding, data collection method, and withdrawals/dropouts [17,18]. Each component is rated as strong (1 point), moderate (2 points), or weak (3 points) based on the tool’s guidelines. Overall assessment of quality, global rating, is rated according to the following criteria: “strong” (1 point) if all components are scored as “strong” or “moderate” (i.e., no “weak” ratings); “moderate” (2 points) if one component is scored as “weak”; and “weak” (3 points) if two or more components are scored as “weak” [17]. Higher score indicates higher risk of bias (poorer quality). Overall, “strong” global ratings indicate a low risk of bias, while “weak” global ratings indicate a high risk of bias. Quality assessments of the included studies were conducted independently by at least two of the current study’s authors. Final decisions on global ratings for all included studies were based on each study’s average global score.

### 2.6. Statistical Analyses

The effect sizes (ESs) for the extracted cognition data (global cognition, executive function, language, learning and memory, and spatial and quantitative) were calculated as the standardized mean difference as detailed in Borenstein et al. (2009) [19]. A positive ES indicated efficacy of Tai Chi in improving cognition as compared to the control condition. If a study measured cognition at multiple time points or under multiple conditions (e.g., single and dual-task), then standardized mean differences and variances were averaged across the different time points or multiple conditions to create the study ES.

In order to confirm the findings of previous systematic reviews and/or meta-analyses, meta-analyses were conducted using a random-effects model to test the effect of Tai Chi in improving cognitive function. A random-effects model was chosen over a fixed-effects model due to the wide variation in experimental factors among the included studies (e.g., Tai Chi dosage, outcomes assessed). Experimental factors can be treated as moderator variables in a meta-analysis. Continuous moderator variables were assessed using meta-regression. Meta-regression analyses of Tai Chi dose (Tai Chi session duration, Tai Chi practice duration per week, study duration, and Tai Chi practice duration for entire study) on study ES were performed to examine the dose–response relationship between Tai Chi and cognitive function. A method-of-moments model for the meta-regression was employed because the effect sizes from the included studies were not normally distributed according to the Shapiro–Wilk test of normality.

All meta-analyses and meta-regressions were conducted using the Comprehensive Meta-Analysis software (version 3.3) (Biostat Inc., Englewood, NJ, USA). A *p*-value < 0.05 was considered statistically significant in all analyses. Effect sizes of 0.2, 0.5, and 0.8 were considered to be small, moderate, and large, respectively. Publication bias in the primary meta-analysis was assessed using a funnel plot of study ES versus standard error.

## 3. Results

### 3.1. Selection of Eligible Studies

**Figure 1:**
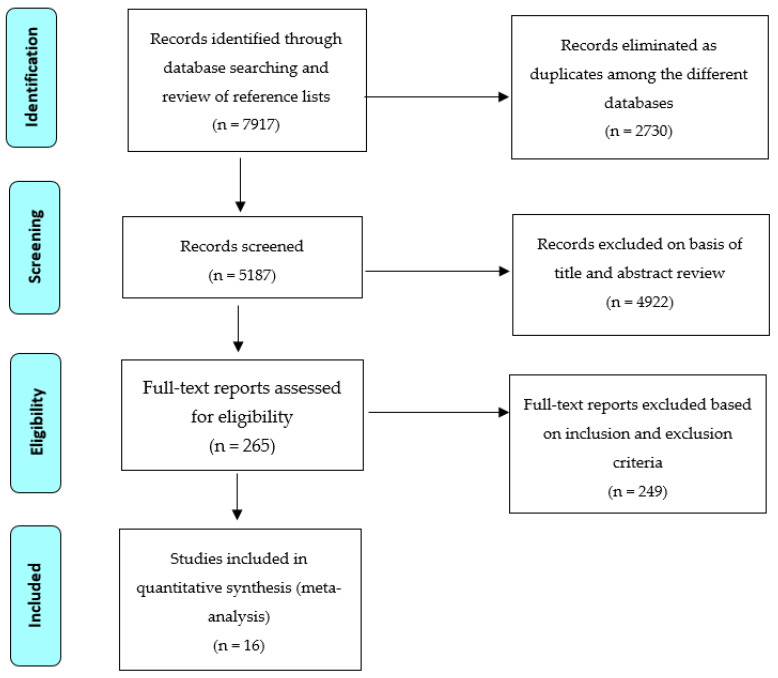
PRISMA flowchart for review and selection of studies in the systematic review.

Figure 1 shows the results of the study selection process. A total of 7917 study reports were originally identified through the database searches and review of article reference lists. During the initial screening phase, 4922 study reports were excluded based on the review of titles and abstracts. After a careful full-text review of 265 study reports, 249 study reports were excluded based on the inclusion and exclusion criteria described above, leaving a total of 16 studies to be included in this systematic review and meta-analysis.

### 3.2. Study Characteristics

The characteristics of the included studies are summarized in the Table 1. In total, 16 studies were included for meta-analysis of the dose–response relationships of Tai Chi on cognitive function in community-dwelling older adults. Among these 16 studies, 14 studies were published as journal articles in peer-reviewed journals. One study (Chu and Lim-Khoo 2014) was published as a conference proceeding in a peer-reviewed journal [20]; one study (Overton-McCoy 2010) was published as a doctoral dissertation [21]. Seven studies used randomized controlled trial (RCT) research design while two used a quasi-experimental research design, and seven studies were cross-sectional studies. In total, there were 1121 subjects; 332 and 789 were males and females, respectively. There were 540 participants in the Tai Chi group; 581 participants in the control group. Mean subject age ranged among the studies from 60 to 77 years. Two studies (Kasai et al. 2010; Sungkarat et al. 2017) involved subjects with mild cognitive impairment (MCI; 92 subjects) [22,23]; 14 studies involved subjects with normal cognition or had no reported cognitive impairment (1029 subjects). Six studies reported using Yang-style Tai Chi; one study (Man et al. 2010) used Ng-style Tai Chi [15], while nine studies did not report the Tai Chi style. Tai Chi doses ranged from a frequency of 1–7 times per week, 20–90 min per session, lasting one month –4.5 years.

### 3.3. Methodological Quality of Included Studies

The methodological quality assessment using the EPHPP Quality Assessment Tool for Quantitative Studies was performed on all 16 studies [17]. Table 2 shows the results of the methodological quality assessment. Two studies (Chu et al. 2014; Overton-McCoy 2010) were judged a global rating of “weak” (high risk of bias) due to blinding, selection bias, or baseline differences between groups for potential confounders [20,21]. The remaining 14 studies were evaluated as “strong to moderate” global rating (average global score 1.0–2.0), indicating a “low to moderate” risk of bias. Overall, the risk of bias was determined to be low.

### 3.4. Meta-Analysis Results

**Figure 2:**
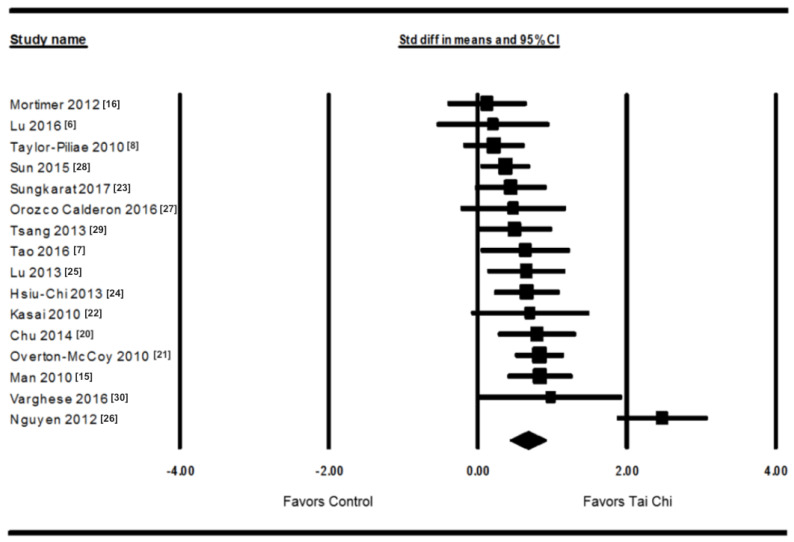
Forest plot of effect sizes (ESs) from the 16 studies that assessed the effect of Tai Chi on cognitive function (Tai Chi group vs. control group).

All included 16 studies in the Figure 2 shows positive, beneficial effects of Tai Chi on cognitive function compared to the control group. Meta-analysis on the 16 studies yielded a statistically significant and moderate-to-large overall ES indicating that there were positive effects of Tai Chi on cognitive function (overall ES = 0.66, *p* < 0.00001; Figure 2).

Table 3 shows the finding of meta-regression analyses examining the dose–response effect of Tai Chi practice on cognitive function. The result indicated that there were no significant relationships between study ES and Tai Chi dose (Tai Chi session duration, Tai Chi practice duration per week, study duration, and Tai Chi practice duration for the entire study) (all *p* > 0.05).

### 3.5. Publication Bias

**Figure 3:**
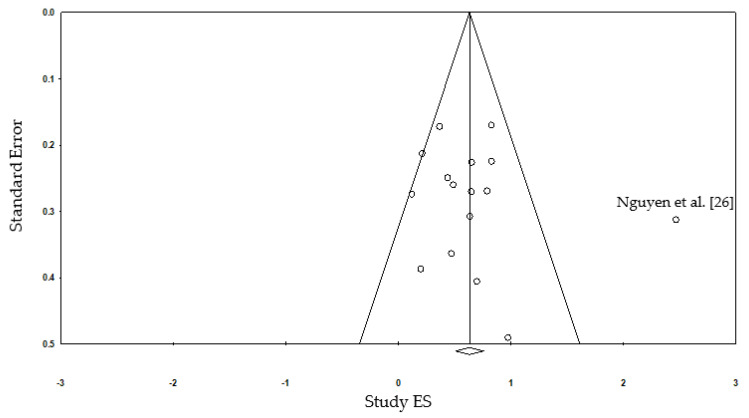
Funnel plot of study effect size (ES) vs. study standard error.

A funnel plot was performed to examine the publication bias (Figure 3). Minor asymmetry was noted in the funnel plot, but was not associated with publication bias. The atypical asymmetry seemed to result mostly from the Ngyuen et al. (2012) study [26]. The result indicated that the risk of publication bias is minimal.

## 4. Discussion

The current study performed a systematic review and meta-analysis and, to our best knowledge, the first to investigate the dose–response relationship of Tai Chi on cognitive function in community-dwelling older adults. This study is different from existing meta-analyses on this topic in terms of the sample and the focus on dose–response research on Tai Chi. Understanding the appropriate dosage for interventions is necessary for informing practitioners and designing future research. The findings of this study were based on a large sample of studies and included research originally published in both Chinese and English. The result of this meta-analysis revealed that older adults who participated in Tai Chi group had greater improvements on cognitive function as compared to those in control group. The findings from this study also indicated that there were no statistically significant relationships between study ES and Tai Chi dose (Tai Chi session duration, Tai Chi practice duration per week, study duration, Tai Chi practice duration for entire study) on cognitive function in community-dwelling older adults.

The results of the methodological quality assessment showed that two studies (Chu et al. 2014; Overton-McCoy 2010) were identified as having a high risk of bias for two or more of the six domains in the EPHPP Quality Assessment Tool for Quantitative Studies [20,21]. If the two studies were excluded from this meta-analysis, it did not alter the effect of the Tai Chi dose on cognitive function. In addition, most studies involved non-random selection and/or did not report the percentage of subjects in the Tai Chi and control groups that agreed to participate in the study before they were assigned to groups. Hence, in the selection bias domain of the methodological quality assessment, one study was evaluated as a high risk of selection bias and 13 studies were evaluated as a moderate risk of selection bias, meaning that participants were somewhat likely or not likely to be representative of the target population [17]. The result of the methodological quality assessment suggests that drawing a random sample from the target population to reduce selection bias is necessary for future research.

In addition, published studies may be affected by selective reporting biases [31]. It is recommended that the grey literature (e.g., doctoral dissertations, conference proceedings) should be included in meta-analyses [32]. Hence, in the present study, one doctoral dissertation (Overton-McCoy 2010) and one conference proceeding (Chu et al. 2014) were included [20,21]. If the two studies were excluded from the analysis, there would still be no statistically significant dose effect of Tai Chi on cognition.

Consistent with existing meta-analyses, this study suggests that Tai Chi has positive effects on cognitive function of community-dwelling older adults. Wayne et al. (2014) conducted a systematic review and meta-analysis to examine the effect of Tai Chi on cognitive performance in older adult population [10]. The results indicated that Tai Chi could enhance cognitive function, especially in executive function. Wu et al.’s (2013) meta-analysis study found that Tai Chi significant improves global cognitive and memory functions, particularly in verbal working memory [11]. Previous systematic reviews also indicated that Tai Chi might improve cognitive abilities in older adults [9,12,13]. Overall, as suggested in previous meta-analysis research, this study confirms that Tai Chi improves cognitive function in community-dwelling older adults.

The results of this study support that Tai Chi is beneficial for preventing cognitive decline and/or improving cognitive health among older adults. There are several potential mechanisms for explaining the positive effects of Tai Chi on cognitive performance. Firstly, hippocampal function is significantly related to cognitive health [7,33,34]. Studies showed that Tai Chi might enhance cognitive function through improvements on hippocampal function and increased hippocampus volumes [7,35,36,37]. Secondly, Tai Chi includes cognitively stimulating activities, such as learning of Tai Chi movement patterns, hand-eye coordination, and memorizing skills, which have been found as a cognitive enhancement on memory, attention, and executive function [10,37,38,39]. Thirdly, Tai Chi involves meditation training, which may positively associate with stress reduction pathways of cognitive improvements [10,40]. Fourthly, Tai Chi is mind–body exercise with the characteristic of aerobic exercise. Aerobic exercise has been found to have positive effects on delaying and/or improving age-related cognitive impairments through increased cerebral blood circulation and neurophysiological pathways [35,41,42,43]. Finally, hypertension and heart disease are related to cognitive decline [1,2]. Studies indicated that Tai Chi has beneficial effects on the control of cardiovascular disease (CVD) risk factors and reduction on blood pressure [5,44,45,46]. It is possible that Tai Chi may benefit cognitive function through reducing CVD risk factors and improving hypertension control.

The key finding of this systematic review and meta-analysis is that there were no statistically significant dose–response relationships between Tai Chi and cognitive function in community-dwelling older adults. This meta-analysis included seven RCTs, two quasi-experimental studies, and seven cross-sectional studies. Causal conclusions from cross-sectional studies may not be drawn; however, if the seven cross-sectional studies were removed from the analysis, there would still be no statistically significant dose–response relationships on cognition. Based on previous studies, a lack of a dose–response relationship for exercise effects is not uncommon. Stagg et al. (2011) found that there was no significant dose–response relationship between the frequency of treadmill exercise training and neuropathic pain [47]. In Polaski et al.’s (2019) meta-analysis study, there were no significant dose intensity effects of exercise (e.g., aerobic, aerobic + strength, aquatic, and strength exercise) on chronic pain [48]. The meta-analysis result from the Northey et al. (2018) study also indicated that longer exercise intervention duration did not predict more beneficial effects on cognitive function in older adults [49]. Sanders et al. (2019) conducted a systematic review and meta-analysis to test the dose effects of exercise (e.g., aerobic, anaerobic, multicomponent and psychomotor exercise) on cognitive function in older adults [50]. The results revealed that exercise was positively related to memory and executive function, but there was no significant dose–response relationship between exercise and cognitive function in older adults without cognitive impairments. Similarly, in this meta-analysis, the results show that there was a positive effect of Tai Chi in improving cognitive function in older adults, but there was no significant dose–response association between Tai Chi dose duration (Tai Chi session duration, Tai Chi practice duration per week, study duration, and Tai Chi practice duration for entire study) and cognition. A longer duration of Tai Chi was unrelated to greater improvements in cognitive function.

One possible explanation for the lack of a dose-dependent effect could be due to potential mediators. Some mediators may potentially influence the dose–response effect of exercise on cognition. For example, hypertension, depression, pain, stress, sleep effectiveness, cardiovascular disease, and self-efficacy may mediate the dose–response relationship between Tai Chi and cognitive function [51]. In addition, in the current study, majority of the subjects (*n* = 1029) did not have cognitive impairments. Additionally, most studies did not focus on sedentary older adults. The Sanders et al. (2019) study revealed that older adults without cognitive impairments had lower exercise effects on cognitive function as compared to older adults with cognitive impairments [50]. Previous studies also indicated that subjects with inactive lifestyles might have greater exercise effects on cognition when compared with subjects with active lifestyles [52,53,54,55]. Overall, subjects’ lifestyles, health status, and cognitive function at baseline may impact the dose–response effects of Tai Chi [51,55]. Future research should consider potential mediators to further advance our understanding of the dose–response relationship between Tai Chi and cognition.

The current study has several potential limitations. First, unpublished literature with non-significant and/or negative results was not available from the electronic databases. Publication bias or systematically unrepresentative of the population of completed studies may occur due to studies with nonsignificant and/or negative findings tend to not be published [56]. Moreover, some confounding factors were not available in the included studies that may affect the dose-dependent effects on cognitive function. A better understanding of physiological and sociopsychological processes of potential mechanisms to explore dose effects of Tai Chi on cognition is necessary for future research in this population. In addition, this study did not include comparative physical activity groups. Future studies with a comparison of exercise groups are recommended to examine if Tai Chi has superior effects on cognitive performance compared to other physical activity interventions. Furthermore, most studies were conducted in Asia. Hence, it may limit the generalizability of the findings due to racial, cultural, and/or geographical differences. Lastly, most studies did not report a detailed Tai Chi protocol, especially Tai Chi style, intensity, instruction methods, and the progression of the Tai Chi program. In the current study, the intensity of Tai Chi for each study was not be able to calculate to examine dose intensity effects due to the lack of intensity information. The complete descriptions of each component of Tai Chi intervention are needed for future research to fully investigate the dose–response effects.

## 5. Conclusions

The results of this study demonstrate that, as compared to the control, Tai Chi improved cognitive performance in community-dwelling older adults, but there were no dose–response effects of Tai Chi on the improvement in cognition. The findings show that Tai Chi had positive effects on cognitive function in older adults, but a longer duration of Tai Chi did not predict larger effects on cognitive function. As many older adults developed cognitive impairments, identifying and testing effective interventions for promoting cognitive health, improving memory functions, and preventing/delaying the onset of dementia for older adults is critically important. This study suggests that Tai Chi has beneficial effects on cognitive function even with a low dose (e.g., three 20-min sessions/week). Future research and clinical practice should consider the duration effects, the styles of Tai Chi, and the dose intensity effects when designing and testing Tai Chi protocols on improving cognitive function for older adults.

## Figures and Tables

**Table 1:** Characteristics of the included Tai Chi studies on the cognitive function in community-dwelling older adults.

Studies/ Locations	Study Design	Study Population	Tai Chi Style	Intervention Modality	Total Tai Chi Dose	Control Group	Cognitive Outcome Measurement
Chu et al. 2014 [20] Philippines	Cross-sectional	I: *n* = 30 I (mean age): 69.30 C: *n* = 30 C (mean age): 64.60 No reported cognitive impairment	Not Specified	At least 30 min a day, 3 times/week for 6 months	2160 min	No exercise	MMSE ^1^ MoCA ^2^
Hung et al. 2013 [24] Taiwan	Quasi experimental	I: *n* = 41 I (mean age): 76.61 C: *n* = 41 C (mean age): 76.29 No cognitive impairment	Not Specified	90 min/session 3 times/week for 12 weeks * including warm up and cool down	1440 min	No exercise	MMSE ^1^
Kasai et al. 2010 [22] Brazil	Non-randomized experimental	I: *n* = 13 I (mean age): 73.54 C: *n* = 13 C (mean age): 75.54 Mild Cognitive Impairment	Yang-style	60 min/session 2 times/week for 13 weeks * including warm up and cool down	1560 min * including warm up and cool down	No exercise	RBMT ^3^ SMC ^4^ DSF ^5^ DSB ^6^
Lu et al. 2013 [25] Hong Kong	Cross-sectional	I: *n* = 28 I (mean age): 73.60 C: *n* = 30 C (mean age): 72.40 No cognitive impairment	Not Specified	Minimum weekly 1.5 h practice for 3 years	14,040 min	No exercise	Auditory Stroop Test Center of Pressure Path (mm)/Area (cm^2^)
Lu et al. 2016 [6] Hong Kong	RCT	I: *n* = 13 I (mean age): 72.80 C: *n* = 14 C (mean age): 67.30 No cognitive impairment	Yang style	60 min/session 3 times/week for 16 weeks	2880 min	No exercise, attended weekly series of music, English, handicrafts and fall prevention classes along with social gatherings	Auditory Stroop Test Center of Pressure Path (mm)/Area (cm^2^) Time to Complete Step-Down Performance
Man et al. 2010 [15] Hong Kong	Cross-sectional	I: *n* = 42 I (mean age): 68.90 C: *n* = 44 C (mean age): 68.20 No cognitive impairment	Ng style	at least 45 min per session, 3 times/week for 3 years	21060 min	No exercise	HKLLT ^7^
Mortimer et al. 2012 [16] China	RCT	I: *n* = 29 I (mean age): 67.3 C: *n* = 24 C2: *n* = 27 C (mean age): 68.2 C2 (mean age): 67.90 No cognitive impairment	Not Specified	20 min/session 3 times/week for 40 weeks	2400 min	C: No exercise C2: Social interaction	Mattis Dementia Rating Scale BNT ^8^ Clock Drawing Test Trail Making Test WAIS-R ^9^ Similarities Test Category Verbal Fluency Test Auditory Verbal Learning Test Stroop Test Rey-Osterrieth Complex Figure Bell Cancellation Test WAIS-R ^9^ Digit Span
Nguyen et al. 2012 [26] Vietnam	RCT	I: *n* = 48 I (mean age): 69.32 C: *n* = 48 C (mean age): 68.73 No cognitive impairment	24-form	30 min/session 2 times/week for 24 weeks	1440 min	No exercise	Trail Making Test
Orozco Calderon et al. 2016 [27] Mexico	Cross-sectional	I: *n* = 17 I (mean age): 70.65 C: *n* = 15 C (mean age): 67.40 No reported cognitive impairment	Yang-style	4 h/week for 4.5 years	Not specified	No exercise	COGNISTAT Test
Overton-McCoy 2010 [21] USA	Cross-sectional	I: *n* = 75 I (mean age): 70.80 C: *n* = 75 C (mean age): 71.31 No cognitive impairment	Not Specified	1–2 times per week; average of 60 min per week for a minimum of one month	Not specified	No exercise	MMSE ^1^ 15-item BNT ^8^ 15-item BNT ^8^ Latency Score
Sun et al. 2015 [28] China	RCT	I: *n* = 72 I (mean age): 68.30 C *n* = 66 C (mean age): 70.10 No cognitive impairment	24-form Yang-style	60 min/session 2 times/week for 6 months * including warm up and cool down	3120 min * including warm up and cool down	No exercise	MMSE ^1^ FAB ^10^
Sungkarat et al. 2017 [23] Thailand	RCT	I: *n* = 33 I (mean age): 68.3 C: *n* = 33 C (mean age): 67.5 Mild Cognitive Impairment	10-form	30 min/session 3 times/week for 15 weeks	1350 min	Health education related to cognitive and fall prevention	Logical Memory Delayed Recall Score; DSF ^5^ DSB ^6^ Block Design Score Trail Making Test Hand Reaction Time
Tao et al. 2016 [7] China	RCT	I: *n* = 21 I (mean age): 62.38 C: *n* = 25 C (mean age): 59.76 No cognitive impairment	24-form Yang-style	30 min/session 5 times/week for 12 weeks	1800 min	Health education	Weschler Memory Scale—Chinese Revision
Taylor-Piliae et al. 2010 [8] USA	RCT	I: *n* = 37 I (mean age): 70.60 C: *n* = 56 C (mean age): 68.20 No reported cognitive impairment	12 short-form Yang-style	Daily practice encouraged for 6 months	Not specified	Health education	Animal-Naming Test DSF ^5^ DSB ^6^
Tsang et al. 2013 [29] Hong Kong	Cross-sectional	I: *n* = 31 I (mean age): 70.30 C: *n* = 30 C (mean age): 72.30 No cognitive impairment	Not Specified	Minimum 90 min/week for 3 years	14,040 min	No exercise	EMG ^11^ Reaction Time EMG ^11^ Movement Time End-Point Accuracy Wrong Movement
Varghese et al. 2016 [30] USA	Cross-sectional	I: *n* = 10 I (mean age): 65.90 C: *n* = 10 C (mean age): 66.40 No cognitive impairment	Not Specified	Minimum 100 h in past year	Not specified	No exercise	Reaction Time Maximal Excursion Value (%) Cognitive Score

Note: I: Intervention group (Tai Chi group); C: Control group; C2: Second control group; RCT: Randomized Controlled Trail; ^1^ Mini-Mental State Examination (MMSE); ^2^ Montreal Cognitive Assessment (MoCA); ^3^ Rivermead Behavioral Memory Test (RBMT); ^4^ Subjective Memory Complaint Scale (SMC); ^5^ Digit Span Forward (DSF); ^6^ Digit Span Backward (DSB); ^7^ Hong Kong List Learning Test (HKLLT); ^8^ Boston Naming Test (BNT); ^9^ Wechsler Adult Intelligence Scale-Revised (WAIS-R); ^10^ Frontal Assessment Battery (FAB); ^11^ Electromyographic (EMG).

**Table 2:** Summary of methodological quality assessment of the included Tai Chi studies.

Studies	Average Component Ratings (1–3 score)						Average Global Rating (1–3 Score)
	Selection Bias	Study Design	Confounders	Blinding	Data Collection Methods	Withdrawals and Dropouts	
Chu et al. 2014 [20]	3.00	2.00	3.00	2.00	1.00	1.33	3.00
Hung et al. 2013 [24]	2.00	1.00	1.00	2.00	1.00	1.00	1.00
Kasai et al. 2010 [22]	2.33	1.00	1.00	2.00	1.00	1.00	1.33
Lu et al. 2013 [25]	2.00	2.00	1.00	2.33	1.00	1.33	1.33
Lu et al. 2016 [6]	2.00	1.00	1.00	2.00	1.00	1.00	1.00
Man et al. 2010 [15]	1.33	2.00	1.00	2.00	1.00	1.00	1.00
Mortimer et al. 2012 [16]	1.67	1.00	1.00	2.00	1.00	1.00	1.00
Nguyen et al. 2012 [26]	2.00	1.00	1.33	2.33	1.00	1.33	1.67
Orozco Calderon et al. 2016 [27]	2.00	2.00	1.00	2.00	1.00	1.33	1.00
Overton-McCoy 2010 [21]	2.00	2.00	3.00	3.00	1.00	1.00	3.00
Sun et al. 2015 [28]	2.33	1.00	1.00	2.00	1.00	1.00	1.33
Sungkarat et al. 2017 [23]	2.00	1.00	1.00	2.00	1.00	1.00	1.00
Tao et al. 2016 [7]	2.00	1.00	1.00	2.00	1.00	2.00	1.00
Taylor-Piliae et al. 2010 [8]	2.33	1.00	1.00	2.00	1.00	1.33	1.33
Tsang et al. 2013 [29]	2.00	2.00	1.00	2.33	2.00	2.00	1.67
Varghese et al. 2016 [30]	2.00	2.00	1.00	2.33	1.00	1.33	1.33

Note: Component Ratings 1: Strong (low risk of bias), 2: Moderate (moderate risk of bias), 3: Weak (high risk of bias); Global Rating 1: Strong (no weak ratings; low risk of bias), 2: Moderate (one weak rating; moderate risk of bias), 3: Weak (two or more weak ratings; high risk of bias).

**Table 3:** Summary of meta-regression analysis examining moderator variables that might explain between-study variance in effect size.

Moderator Variable	Slope	*p*-Value
Tai Chi session duration (min) (12 studies)	−0.0025 min^−1^	0.8115
Tai Chi practice duration per week (min) (including warm-up and cool-down) (15 studies)	−0.0020 min^−1^	0.2380
Study duration (weeks) (15 studies)	−0.0005 wk^−1^	0.7913
Tai Chi practice duration for entire study (min) (16 studies)	−0.0000 min^−1^	0.6588

## Data Availability

The data supporting this meta-analysis were extracted from previously published studies, which have been cited in this paper.
